# Dismantling the myth of “all foods fit” in eating disorder treatment

**DOI:** 10.1186/s40337-024-01017-9

**Published:** 2024-05-17

**Authors:** Timothy D. Brewerton, Kim Dennis, David A. Wiss

**Affiliations:** 1 Department of Psychiatry and Behavioral Sciences, Medical University of South Carolina, Charleston, SC USA; 2 SunCloud Health, Chicago, IL USA; 3 Department of Psychiatry, University of Illinois Chicago College of Medicine, Chicago, IL USA; 4 Nutrition in Recovery, Los Angeles, CA USA

**Keywords:** Eating disorders, Nutritional rehabilitation, Food allergies, Food addiction, Ultra-processed food, Treatment

## Abstract

We call for a reevaluation of the long-standing dogmatic nutritional principle that “all foods fit” for all cases of eating disorders (EDs) and its corollary, “there are no bad foods” (for anyone ever) during ED treatment. Based on accumulated scientific research, we challenge these ideologies as outdated, confusing, and potentially harmful to many patients. We review the evidence that indicates the folly of these assumptions and show there are a variety of exceptions to these rules, including (1) food allergies, sensitivities, and intolerances, (2) religious and spiritual preferences or doctrines, and (3) the ubiquitous emergence and widespread availability of ultra-processed foods leading to the potential development of addiction-like eating and a higher prevalence of various medical and psychiatric comorbidities, as well as higher mortality. This evidence supports a nutritional psychiatry approach that should be integrated into (rather than dissociated from) ED treatment research and practice.

## Comment

The adage, “all foods fit,” has been an eating disorder (ED) treatment dogma for decades. In other words, there is an almost monolithic belief or proclamation within the ED field that “there are no bad foods” when it comes to the nutritional rehabilitation of all patients with all forms of EDs. Decades of clinical experience have demonstrated that this approach is helpful in addressing the restrictive dimension of ED pathology across phenotypes. However, for many individuals with EDs, eating symptoms extend beyond the domain of dietary restraint. The well-intentioned assertion that “all foods fit” persists in the anti-diet culture movement despite mounting evidence substantiating the association of ultra-processed foods with negative physical and mental health outcomes.

This essay calls into question these suppositions and proclaims that for many patients, they, at best, represent an incomplete truth and, at worst, stand to cause harm. When uniformly and rigidly applied to all patients, the age-old adage that “all foods fit” no longer serves the entire ED treatment community. This is most pressingly true for patients with EDs from communities historically marginalized and underrepresented in ED research, advocacy groups, training programs, and treatment settings. We will discuss why assumptions associated with “all foods fit” are outdated and incomplete concepts for some patients with EDs. These strong assumptions no longer align with the evidence base that has emerged over the last decade, which we as clinical scientists strive to translate to good clinical care.

“Food is medicine,” a shortened version of “Let thy food be thy medicine, and thy medicine be thy food,” has also been a related ED dogma for decades. This saying (often misattributed to Hippocrates), carries a lot of truth and heuristic value [1]. However, are all foods medicinal? Are there any “bad” or harmful medicines for any given patient? Of course, there are. Medicines are neither “good” nor “bad” unto themselves. It’s how they’re used or prescribed, for which problems, in which persons, at what times, and in what doses. The same medicine may help or harm an individual depending on many factors, and biopsychosocial-spiritual context is paramount.

The scientific literature clearly indicates that some foods “don’t fit” and *are* harmful, at least to some people under specific circumstances. An obvious example is a food allergy, which can cause various problems, including anaphylaxis, which has been increasing in prevalence and affects up to 20% of individuals in the population [2, 3]. There are 14 identified food allergens: celery, cereals containing gluten (such as barley and oats), crustaceans (such as prawns, crabs, and lobsters), eggs, fish, lupin, milk, mollusks (such as mussels and oysters), mustard, peanuts, sesame, soybeans, sulfur dioxide and sulfites (at concentrations > ten parts per million) [4]. Food allergies are associated with high degrees of psychological distress and significantly reduced health-related quality of life [5]. In addition, recent studies suggest positive associations between EDs and food allergies, so this is an important area of inquiry in clinical evaluation and subsequent dietary management [6].

A related problem is food sensitivity, in which certain individuals develop various adverse effects, both acute and chronic, including neurobehavioral, gastrointestinal, metabolic, or teratogenic consequences, upon exposure to certain substances, such as gluten, food additives, Fermentable Oligo-, Di- and Mono-saccharides And Polyols (FODMAPs), and non-nutritive sweeteners [7–13]. There are several examples of these phenomena relevant to the treatment of patients with EDs, including comorbid migraine headaches, irritable bowel syndrome, attention deficit hyperactivity disorder (ADHD), major depressive disorder, and anxiety disorders [12, 14–25], in which a nutritional psychiatry perspective can be highly influential [26–28].

While not eating can be a trigger for acute migraine headache, so can the ingestion of certain foods, such as those containing monosodium glutamate, sugar, alcohol, nitrites, gluten, cheese, chocolate, caffeine, and fermented, processed, and pickled foods [29–31]. However, not all foods will trigger migraine in every individual with migraines, and there is tremendous variation that needs to be individually evaluated [32]. For a patient with an ED, restrictive or not, and comorbid migraine, who routinely gets migraine headaches after consuming certain food types, they may need a meal plan that explicitly does not include or includes very little of those foods. Often, in ED treatment, patients expressing preferences to avoid these foods are assumed to be related to restrictive pathology rather than intuitive eating and adaptive dietary restraint. Nevertheless, dietary interventions have been successfully employed in the treatment of this common comorbidity that needs to be routinely considered in the comprehensive treatment of ED patients [13, 30, 33].

Attention deficit hyperactivity disorder (ADHD) is another disorder commonly associated with EDs that may be affected by nutritional intake of specific substances, such as food color additives [34–37]. Of particular interest are several studies showing that a diet high in refined sugar and saturated fat can increase the risk of ADHD, whereas a diet characterized by higher consumption of whole fruits and vegetables may be protective against ADHD [38].

Apart from allergies and sensitivities, some individuals have food intolerances (e.g., lactose, gluten, onions, shallots, leeks, bell peppers, etc.), all of which can cause gastrointestinal distress and which have been associated with anxiety and depression [39–43].

Then, there are individual food preferences for religious, spiritual, and/or ethical reasons, such as those who are vegan, vegetarian, pescatarian, Kosher, etc [44–46]. . . These proclivities have only sometimes been respected by ED therapists and programs, and practices have evolved over time as it has been realized that more research is needed to decipher their effects on clinical course and outcomes [46–49].

What is particularly controversial of late is the issue of food addiction (FA) or ultra-processed food addiction (UPFA), which, despite its strong scientific foundation [50, 51], remains highly controversial in ED circles [52]. Unfortunately, UPFA and its treatment often get conflated with caloric restriction and diet culture, which are not equivalent. In fact, there are studies currently examining flexible, calorically robust, and weight-neutral dietary interventions for patients with FA [53]. Ultra-processed foods (UPFs), which are now identified as NOVA-4 in the NOVA classification system [54], have been associated with a host of adverse health effects and increased mortality [55–65]. The NOVA classification system categorizes foods according to their degree of processing, and the NOVA-4 group includes foods with the highest degree of processing. Specifically, they include packaged formulations of industrial ingredients and substances derived from foods or else created in laboratories, and typically contain little or even no whole foods [66]. UPFs have been shown to promote binge eating [67, 68] and higher body weights [69–72], as well as worsen depression [73–77], type-2 diabetes [78, 79], cardiovascular disease [80–84], and chronic kidney disease [85]. In terms of mechanisms for this multitude of adverse effects, UPFs have been associated with stimulating inflammation, altering multiple neurobiological and endocrine pathways (e.g., insulin), and disrupting the microbiome [86–88]. The presence of UPFA has also been associated with a poorer treatment outcome for binge eating disorder [89], which is known to be highly comorbid with addiction-like eating [90–92].

The intake of UPFs accounts for the great majority of calories ingested in Westernized countries and is only increasing in availability and popularity as time goes on [93]. Estimates of the worldwide prevalence of FA/UPFA using the Yale Food Addiction Scale are on the order of 14% in non-clinical samples [94]. In addition to having high concentrations of refined sugars and oils, UPFs also contain potentially obesogenic endocrine-disrupting chemicals that have been added (or neo-formed during high heat processes) to foods and food packaging by the food industry for the last several decades [95–106]. Many have called out the crisis in food quality as a major public health problem [65, 107], and its contribution to the ED field is substantial, albeit mostly unrecognized. Up to 80% of food advertising promotes UPF and drink products, with food companies often targeting communities of color [108]. The ED community disregarding the role of the food supply chain and systemic racism in food access further compounds the racial inequities marginalized communities face with regard to accessing culturally attuned, comprehensive ED care [109].

We contend that inattention to and outright avoidance of the adverse effects of the Western diet has been a gaping blind spot of ED research and practice and, therefore, draw attention to the dire need to address this [86]. Evidence-based treatment of EDs is a work in progress, and much research remains to be done to establish best practices, particularly for patients of color and for newer diagnostic categories like atypical anorexia nervosa. Outcomes are less than ideal, particularly in complex patients with multimorbidity, such as those with addiction-like eating [110, 111].

We understand the resistance to any departure from an “all foods fit” philosophy. The toxic impact of diet culture, intensified by social media, and its impact on people with restrictive EDs of all body sizes is an important factor. A “one size fits all” mentality is simple and easy to implement, particularly in treatment centers where services are scaled, staff turnover may be high, and individualized nutritional approaches may be challenging to implement and manage in a therapeutic milieu. Maintaining the status quo self-serves ED programs, which then don’t have to individualize nutritional approaches and can avoid the clinical work of supporting patients who may be triggered by peers with different nourishment plans. This stance may avoid potential conflicts among patients with various types of EDs who have different nutritional needs. Patients with ED (and their care teams) are often competitive, prefer rigid rules, and have strong tendencies to compare treatment approaches, including meal plans, with each other or across programs.

We posit that another major reason for the anti-FA stance of many in the ED field is that FA/UPFA is associated with obesity. This topic has become taboo in the ED field largely as a result of weight-based stigma that has harmed (and continues to harm) many patients with ED living in larger bodies navigating medical and mental health treatment settings [112–116]. Any suggestion of reducing or eliminating UPFs is often equated with caloric restriction or attempts to lose weight, which is viewed as anathema to a traditional ED treatment philosophy [117]. All too often, ED professionals do not acknowledge that there *may* be times when a patient’s desire to lose weight is not necessarily reflective of restrictive ED pathologies, such as when there is comorbid type II diabetes, pseudotumor cerebri, hypertension, heart disease, or severe osteoarthritis. In fact, there are *definitely* times when weight loss occurs as a result of improved eating behaviors and normalized eating in the course of ED treatment. There may be times when it is indicated and appropriate, even in patients who may have or have had EDs, to support rather than pathologize a patient’s desire to lose weight, for example, when the benefits of weight loss can reasonably be expected to outweigh the risks. Care must be taken to mitigate the risk of worsening disordered eating behaviors and cognitions and to ensure that patients are fully nourished. The importance of advocacy for body neutrality and weight-inclusive care is essential, especially for ED patients in larger bodies who have suffered great harm from implicit bias related to weight stigma. Ongoing advocacy is undoubtedly of critical importance, but we maintain that a balanced, culturally attuned, individualized, and scientifically sound approach is needed, given how highly contentious these issues are and the limitations of these data. For example, recent studies illustrate that weight loss can be achieved in patients in higher weight categories who are receiving appropriate medical supervision without exacerbating ED symptoms and behaviors [118–123]. In fact, in a recent systematic review of ED risk during behavioral weight management in adults with overweight or obesity, out of 14 studies that reported the prevalence of binge eating, all 14 studies reported a reduction in binge frequency [121]. In addition, in those studies that measured global ED symptoms, the majority of studies reported reductions in ED risk. As stated in the new guidelines for treating EDs in patients living in larger bodies, “The presence of an eating disorder should not delay and does not preclude treatment for other medical/psychological conditions” [117]. Nevertheless, future research on better meeting the needs of the increasing population of ED patients with higher weights will hopefully continue to open up more avenues of effective, weight-inclusive, comprehensive care [124].

Notwithstanding, if the ED treatment community doesn’t acknowledge the evolving nutritional science, the role of the food environment, and medical multimorbidity and integrate this biopsychosocial perspective into our practices, there is a great risk of inflicting harm on our patients, as well as losing our credibility as a field [86]. In our opinion, it is imperative that ED clinicians challenge any all-or-nothing thinking that they may have when it comes to the dogmatic nutritional approaches like “all foods fit” and instead make way for a more nuanced, scientifically sound approach that accounts for the myriad of complex comorbidities that ED patients may present with that may necessitate more fine-tuned nutritional prescriptions. We owe it to the 30–40% of patients who do not fully recover with current evidence-based, standard ED treatment approaches, not to mention the countless patients who have no access to clinical trials or treatment settings and of course, those who avoid ED care altogether for fear of being pathologized and poorly served by paternalistic ED treatment delivery models.

## Data Availability

No datasets were generated or analysed during the current study.

## Declarations

### Clinical implications

The ED dogma that “all foods fit” is outdated and potentially harmful to many. Evidence indicates several exceptions to this rule, including the following: (a) food allergies, sensitivities, intolerances, (b) religious/spiritual preferences (c) ultra-processed foods, which promote addiction-like eating, higher morbidity/mortality. This evidence should be integrated into (rather than dissociated from) ED treatment.
